# Assessment and Performance of Pooled Serum Samples for Monitoring Farm-Level Immunity in Tilapia Infected with Tilapia Lake Virus

**DOI:** 10.3390/v17070877

**Published:** 2025-06-22

**Authors:** Jidapa Yamkasem, Puntanat Tattiyapong, Ian A. Gardner, Win Surachetpong

**Affiliations:** 1 Department of Veterinary Microbiology and Immunology, Faculty of Veterinary Medicine, Kasetsart University, Bangkok 10900, Thailand; jidapa.yam@ku.th (J.Y.); been_best@yahoo.com (P.T.); 2 Atlantic Veterinary College, University of Prince Edward Island, 550 University Avenue, Charlottetown, PE C1A4P3, Canada; iagardner@upei.ca; 3 Laboratory of Biotechnology, Chulabhorn Research Institute, Bangkok 10210, Thailand

**Keywords:** TiLV, immunity, ELISA, pooling, serum samples, tilapia, analytical sensitivity, monitoring

## Abstract

Effective surveillance of viral disease in fish populations is critical for disease control and the sustainable development of global aquaculture. Here, we evaluated the application and performance of pooled serum samples using an indirect ELISA based on recombinant segment 4 protein to assess farm-level immunity in tilapia infected with Tilapia lake virus (TiLV). The TiLV-S4 ELISA was developed using a recombinant nucleoprotein (segment 4) antigen, optimized through checkerboard titration, and validated for repeatability and reproducibility, with intra- and inter-assay coefficients of variation below 10%. A pooling strategy was used to combine multiple serum samples before testing for the presence of TiLV-specific antibodies using an enzyme-linked immunosorbent assay (ELISA). Our results showed that pooling five serum samples was effective for detecting TiLV-specific antibodies, particularly when multiple seropositive individuals were presented in the pool, supporting its application for population-level surveillance. However, ELISA sensitivity may be reduced when only one seropositive sample is included in the pool, due to the dilution effects. Despite this limitation, pooled testing yielded a high proportion of positive results, suggesting similar detection performance in many cases. Overall, the pooling strategy provides a cost-effective and time-efficient approach for large-scale monitoring of immune status in tilapia populations.

## 1. Introduction

Tilapia lake virus (TiLV) is an important pathogen affecting various species of tilapia and causing substantial economic losses in many countries [1,2]. TiLV outbreaks can lead to mortality rates as high as 90%, which poses a severe threat to the sustainability of tilapia farming [3]. Accurate and reliable diagnostic methods are crucial for mitigating these losses and controlling the spread of TiLV. Various techniques have been established to detect TiLV in infected fish samples, including quantitative polymerase chain reaction (qPCR) assays, immunohistochemistry, in situ hybridization, and loop-mediated isothermal amplification methods [4,5,6,7]. Although these assays are highly sensitive and specific, they primarily focus on detecting genomic material of the virus. These approaches make it challenging to determine the TiLV-exposure status of individual fish and populations at a later stage of an outbreak or after the outbreak has ceased.

Serological methods such as enzyme-linked immunosorbent assays (ELISAs) offer a more cost-effective and practical approach for evaluating the immune response in hosts. The technique also serves as a rapid alternative for large-scale screening of samples. For TiLV, previous reports indicate that fish exposed to the virus can rapidly mount specific antibody production, with these antibodies persisting for more than four months [8]. This evidence led to the effort to develop ELISAs to assess the serum antibody concentrations using whole viral antigen [8], a recombinant protein from segment 8 [9] and segment 4 [10] of TiLV. These assays represent a significant advancement in TiLV diagnostics and can be further developed into effective tools to monitor infection status in tilapia farms. In other fish species, serological techniques such as ELISA have been applied to monitor immune responses against different viruses, such as Koi Herpes Virus, Grass Carp Reovirus, and Viral Hemorrhagic Septicemia [11,12,13]. Additionally, the technique can be applied to evaluate vaccine efficacy and for disease surveillance within fish farms [14,15,16].

However, testing individual fish serum samples is costly and time-consuming. The pooling technique, which combines multiple individual samples into a single pooled sample, presents a cost-effective and more practical approach for laboratory practices [17,18,19]. Therefore, studying the application of pooled serum samples to assess TiLV status within tilapia populations could significantly enhance the efficiency of monitoring the TiLV infection status of farms. While pooling techniques have been successfully used to detect TiLV genomic material in fish tissues [20], their analytical sensitivity and applicability to immunological assays have not yet been fully assessed.

The aim of this research was to develop an indirect ELISA based on a recombinant protein derived from TiLV segment 4, a structural nucleoprotein with strong antigenic properties and considered a promising target for immunological studies [8]. The assay was designed to detect TiLV-specific immunity in fish and to evaluate the sensitivity and performance of pooled serum samples. The performance of different pooling strategies was compared with individual serum samples to assess their diagnostic efficacy. This study provided important insights into the feasibility of using pooled serum samples as a diagnostic tool for TiLV in tilapia farms.

## 2. Materials and Methods

### 2.1. Serum Samples and Ethical Approval

A total of 320 fish serum samples were used in this study. All samples were stored at −20 °C until used. Based on their source and exposure to TiLV, samples were divided into two groups: laboratory challenge samples and field study samples. Sera from red hybrid tilapia (*Oreochromis* spp.) were obtained from previous experimental studies conducted in the animal facility at the Faculty of Veterinary Medicine, Kasetsart University, Thailand from 2019 to 2021. These samples included 40 serum samples from fish experimentally infected with TiLV strain KUTV08 (TiLV-seropositive) and 120 serum samples from control fish (TiLV-seronegative). The presence or absence of immunoglobulin M (IgM) serum antibodies against TiLV in these samples was previously examined using an in-house protocol of the enzyme-linked immunosorbent assay (ELISA) with whole TiLV virus as the antigen (hereafter named TiLV-WV-ELISA) [8]. In addition, 160 serum samples were collected in 2022 from four tilapia farms with a history of TiLV outbreaks in Kanchanaburi province, Thailand. Details of samples are provided in Table 1. Blood was collected from red hybrid tilapia, ranging in size from 30 to 400 g, in farms with TiLV outbreaks that occurred 28 to 90 days after first TiLV mortality.

**Figure 1:**
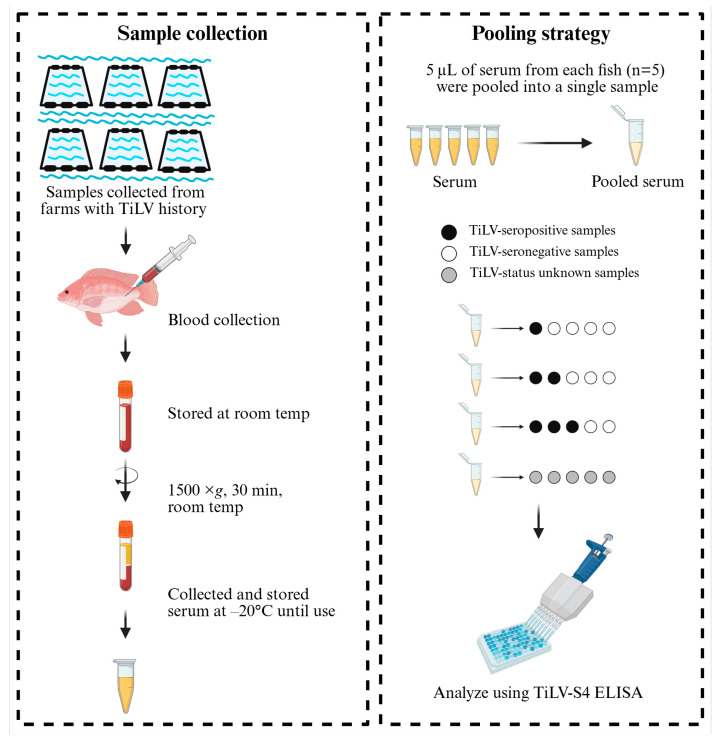
Sample collection and pooling strategy. (Left) Blood samples were collected from farms with a history of TiLV outbreaks. Blood was stored at room temperature and then centrifuged at 1500× *g* for 30 min at room temperature. Serum was collected and stored at −20 °C until analysis. (Right) For the pooling procedure, 5 µL of serum from each of five individual fish were combined into a single pooled sample. Pooled serum samples, which included TiLV-seropositive, TiLV-seronegative, and TiLV-status unknown samples, were then analyzed using the TiLV-S4 ELISA.

All blood samples were collected in non-anticoagulation tubes for serum separation and were centrifuged at 1500× *g* for 30 min at room temperature. Serum was collected in 1.5 microcentrifuge tubes for antibody detection and stored at −20 °C until further analysis (Figure 1). This study was approved by the Animal Ethics Committee of the faculty of Veterinary Medicine, Kasetsart University, Bangkok, Thailand. All animals used in this study were handled in accordance with the Animal Ethics procedures and guidelines (protocol number ACKU65-VET-088).

### 2.2. Preparation of Recombinant TiLV Segment 4 Protein

#### 2.2.1. Construction of Recombinant TiLV Segment 4 Plasmid

The sequence encoding TiLV segment-4 was obtained from the NCBI GenBank database (accession number KX631924.1). To enhance protein expression and simplify purification, codon optimization was performed, and a His-tag was added to the C-terminal end. The Compute pI/MW online tool (https://web.expasy.org/compute_pi/ accessed on 25 March 2022) was used to predict the molecular weight of optimized protein. The optimized TiLV-S4 gene was synthesized by Synbio Technologies, LLC (Monmouth Junction, NJ, USA) and cloned into the pET-28a(+) expression vector using the restriction enzymes NcoI and XhoI. The ligation reaction was carried out using T4 DNA ligase.

#### 2.2.2. Expression and Purification of Recombinant Protein

The pET-28a-TiLV-S4 was transformed into *Escherichia coli* BL21-competent cells. Transformed bacteria were incubated with isopropyl-β-D-thiogalactoside (IPTG) to stimulate expression of TiLV-S4. Briefly, positive transformants were cultured in Luria–Bertani medium containing 50 µg/mL of kanamycin at 37 °C with shaking at 225 rpm and induced with 0.2 mM IPTG when the optical density at 600 nm (OD_600_) of culture material reached 0.5. Eighteen hours post-induction at 18 °C, bacteria were harvested and lysed using ultra-sonication (5 s on, 7 s off, total time 4 min) on ice. Following centrifugation (10,000× *g*, 30 min, 4 °C), the supernatant of bacterial lysates was collected and analyzed through SDS-PAGE followed by Coomassie blue staining. For Western blotting, proteins were transferred onto a polyvinylidene fluoride membrane (Bio-Rad, Hercules, CA, USA) using semi-dry transfer system (Bio-Rad, Hercules, CA, USA). Membranes were blocked in 3% Bovine serum albumin (Sigma, St. Louis, MO, USA) in PBS-T (PBS with 0.1% Tween-20) overnight and then incubated at 4 °C with rabbit polyclonal anti-TiLV serum (generated by immunizing rabbits with purified whole TiLV antigen) [8], diluted 1:1000. After washing, membranes were incubated with HRP-conjugated anti-rabbit IgG (SeraCare, Milford, MA, USA), followed by detection using a LumiFlash™ Infinity Chemiluminescence substrate (Visual Protein, Taipei, Taiwan) and imaging on a ChemiDoc™ system (Bio-Rad, Hercules, CA, USA). The TiLV-S4 protein was purified from supernatant using a HisTrap™ HP (Cytiva, Amersham, UK) with an ӒKTA pure™ chromatography system (Cytiva, Amersham, UK) according to manufacturer’s instructions. Eluted protein solution was concentrated using a Nanosep^®^ Centrifugal Filter with Omega membrane 10 K (Pall Corporation, Port Washington, NY, USA), and final protein concentration was determined using a Pierce BCA Protein Assay Kit (Thermo Fisher Scientific, Waltham, MA, USA).

### 2.3. Optimization of TiLV-S4 ELISA

The working concentrations of ELISA reagents, including the antigen and primary antibody were determined by checkerboard titration. The optimal concentrations for the antigen and primary antibody were selected based on the highest OD_450_ ratio of the positive sample to the negative sample (P/N ratio). Ninety-six-well ELISA plates (Thermo Fisher Scientific, Waltham, MA, USA) were coated with 100 µL of TiLV-S4 protein antigen (0, 0.5, 1, 2, and 5 µg/mL) diluted in 1X KPL coating solution (SeraCare, Milford, MA, USA) and incubated overnight at 4 °C. Plates were then blocked with 200 µL of 3% (*w*/*v*) bovine serum albumin (BSA) (Sigma-Aldrich, St. Louis, MO, USA) in PBS containing 0.05% Tween-20 (0.05% PBS-T) for 1 h at room temperature. Following the blocking step, buffer was discarded, and plates were washed three times with 200 µL of 0.05% PBS-T for 5 min each. Positive and negative serum samples, obtained from archived infection studies as described in Section 2.1, were diluted in 0.05% PBS-T containing 3% BSA to final concentrations of 1:100, 1:200, 1:400, 1:800, 1:1600, 1:3200, and 1:6400. These samples were added to wells, incubated for 1 h at room temperature, and subsequently washed three times. Next, 100 µL of secondary antibody (anti-tilapia IgM monoclonal antibody) (Aquatic Diagnostics Ltd., Stirling, UK) diluted 1:1000 in 0.05% PBS-T containing 3% BSA was added to each well and incubated for 1 h at room temperature to detect IgM against TiLV in serum samples. Plates were washed three times with 0.05% PBS-T, followed by incubation in 100 µL anti-mouse IgG HRP-conjugated antibody (SeraCare, Milford, MA, USA) at a dilution of 1:2000 in 0.05% PBS-T containing 3% BSA. Plates were incubated at room temperature for 1 h. After incubation, plates were washed three times, and 50 µL of 3,3′,5,5′-tetramethylbenzidine (TMB) substrate solution (Thermo Fisher Scientific, Waltham, MA, USA) was added to each well for the chromogenic reaction, which was carried out at room temperature for 20 min in the dark. The reaction was terminated using 50 µL of 2M sulfuric acid, and optical density at 450 nm (OD_450_) was measured using a microplate reader and analyzed using Gen5 version 3.0 software (Biotek, Winooski, VT, USA).

### 2.4. Cut-Off Value for Classification of an ELISA Result as Positive or Negative

To determine the cut-off value for the TiLV-S4 ELISA, 120 TiLV-seronegative samples collected from farms with no history of TiLV infection were tested. Each sample was analyzed in triplicate, and the mean OD_450_ value was used for analysis. The percentage reactivity (PR) value for each sample was calculated using the following formula:

PR value = [(OD value of tested sample − negative control) ÷ (OD value of positive control − negative control)] × 100%.

The PR cut-off value for the serum-based ELISA was determined by calculating the mean PR value ($\overset{-}{x}$) of 120 TiLV-seronegative samples plus 3 standard deviations (SD). A sample was considered positive if its PR value was equal or greater than the cut-off value, otherwise the sample was considered negative. 

### 2.5. Repeatability and Reproducibility of TiLV-S4 ELISA

To evaluate the repeatability (intra-assay variation) and reproducibility (inter-assay variation) of the TiLV-S4 ELISA, a total of six serum samples were selected including three TiLV-seropositive samples (with previously determined OD values of 1.07, 1.45, and 1.93 using the whole-virus ELISA) and three TiLV-seronegative samples (with OD values of 0.20, 0.25, 0.59) were selected. For repeatability assessment, each sample was tested in triplicate within a single ELISA run. For reproducibility assessment, each sample was tested once per run across three independent ELISA runs conducted on three different days. The mean, standard deviation (SD), and coefficient of variation (CV%) were calculated for each sample under both intra- and inter-assay conditions to determine the consistency and robustness of the assay.

### 2.6. Sensitivity and Specificity of TiLV-S4 ELISA

Point estimates of diagnostic sensitivity and specificity of the TiLV-S4 ELISA, along with 95% exact binomial confidence intervals (CI), were estimated by comparing the results to those of 40 TiLV-seropositive and 40 TiLV-seronegative serum samples obtained from controlled laboratory challenge studies. These samples had been previously tested using the TiLV-WV-ELISA, which was used as the reference method for evaluating the performance of the TiLV-S4 ELISA. The PR cut-off value of the TiLV-S4 ELISA was calculated as described in Section 2.4, and was used to classify results as TiLV-S4-positive or -negative for calculation of diagnostic sensitivity and specificity.

### 2.7. Viral Antigen Preparation and TiLV-WV ELISA Protocol

The protocol established by Tattiyapong (2020) [8] was applied for preparation of whole protein for the TiLV-WV ELISA assay. Briefly, TiLV strain KUTV08 was propagated by passaging in E-11 cells (ECACC, Salisbury, UK), and virus was harvested five days post-inoculation. Viral stock was then concentrated and purified. The concentration process involved passing virus through a 30% sucrose cushion at 107,000× *g* for 90 min at 4 °C using an Optima MAX-XP Ultracentrifuge (Beckman Coulter, Brea, CA, USA). Subsequently, the pellet was re-suspended in TN buffer and further purified through a sucrose gradient concentration of 30%, 40%, and 50% (*W*/*V*) at 107,000× *g* for 120 min at 4 °C. The pellet was collected from the 40% and 50% sucrose fractions, re-suspended in TN buffer, and stored at −80 °C for further analysis.

For the TiLV-WV ELISA assay, working concentrations and dilution ratios were as follows: viral antigens at 2.5 µg/mL, primary antibody (fish serum sample) diluted at 1:100, secondary antibody (anti-tilapia IgM monoclonal antibody) diluted at 1:1000, and tertiary antibody (HRP-conjugated anti-mouse IgG antibody) diluted at 1:2000 [8]. The ELISA protocol followed the same steps as described in Section 2.3.

### 2.8. Design of Pooling Experiments

Serum samples from individual farm fish, as described in Section 2.1, were used to evaluate the efficiency of TiLV-S4 ELISA with pooled samples. The diagnostic accuracy of pooling was assessed by selection of one, two, or three positive serum samples mixed with four, three, and two negative serum samples, respectively (Figure 1). To create a pool of five samples, 5 µL of each serum sample was aliquoted from five individuals and mixed into a 1.5 mL microcentrifuge tube. After pooling, serum samples were processed for detection of antibodies against TiLV using the new TiLV-S4 ELISA assay (Figure 1).

### 2.9. Data Management and Statistical Analysis

Pooled and individual TiLV-S4 ELISA results from each farm were combined. The individual TiLV-S4 ELISA result for each sample used in the pool was considered the reference standard (classified as positive if the PR value was >37.11, and negative otherwise). The corresponding pooled result was then classified as either a true positive, true negative, or false negative for each sample set. No false positives were obtained. For positive pools, the median and range of individual positive samples were calculated. Additionally, the ratio of the mean PR% of the 5 individual sample pools to the corresponding pooled PR was calculated. This value was expected to be approximately one in the absence of technical errors or antibodies aggregation/clumping. Frequencies were compared by chi-square or Fisher exact tests, and distributions of PR ratios between Farm A and Farms B, C, and D were compared with a Mann–Whitney rank sum test. *p* values < 0.05 were considered statistically significant.

## 3. Results

### 3.1. Production of TiLV-S4 Protein and Its Specificity

**Figure 2:**
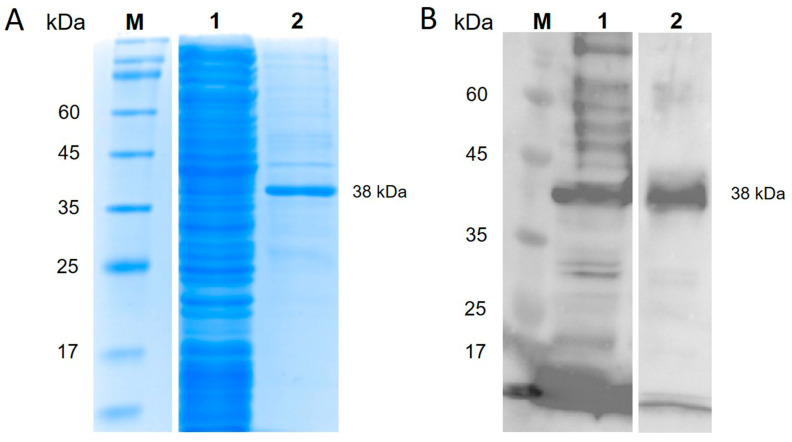
Expression and purification of TiLV-S4 protein. (**A**) SDS-PAGE analysis stained with Coomassie blue showing the expressed TiLV-S4 protein. (**B**) Western blot of the TiLV-S4 protein with rabbit anti-TiLV antibody. Marker (Lane M), total protein (Lane 1), and purified TiLV-S4 protein (Lane 2).

Recombinant TiLV-S4 protein was produced from *E. coli* BL21 and purified using a HisTrap™ HP column and affinity chromatography. After separation on SDS-PAGE, a distinct 38 kDa band corresponding to the TiLV-S4 protein was detected (Figure 2A). Further analysis by Western blot, using rabbit serum collected from animals exposed to TiLV, confirmed the specificity of the protein by showing a specific interaction at this band size (Figure 2B).

### 3.2. Development and Optimization of a TiLV-S4 ELISA

#### 3.2.1. Validation of TiLV-S4 Recombinant Protein and Primary Antibody Concentrations

To develop the TiLV-S4 ELISA for detecting TiLV-IgM antibodies, a checkerboard titration was conducted to optimize assay conditions. Optimization criteria included ensuring a high OD_450_ value for positive serum and a low value for negative serum, and maximizing the positive-to-negative (P/N) ratio. The optimal concentration of TiLV-S4 protein for coating ELISA plates was determined to be 0.5 µg/mL (0.05 µg/well). Primary antibodies (fish serum samples) were used at a working dilution of 1:100, the secondary antibody (anti-tilapia IgM antibody) at 1:1000, and the tertiary antibody (HRP conjugated anti-mouse IgG antibody) at 1:2000.

#### 3.2.2. Repeatability and Reproducibility of the TiLV-S4 ELISA

To validate the repeatability and reproducibility of the TiLV-S4 ELISA, six serum samples (three TiLV-seropositive and three TiLV-seronegative) were tested. Detailed results are presented in Table 2, which show intra-assay CVs ranging from 1.1% to 8.7%, and inter-assay CVs ranging from 1.7% to 7.7% (Table 2). These results indicate that the new TiLV-S4 ELISA demonstrated good repeatability and reproducibility.

#### 3.2.3. Determination of the Cut-Off Value for TiLV-S4 ELISA for Classification of Results as Positive or Negative

**Figure 3:**
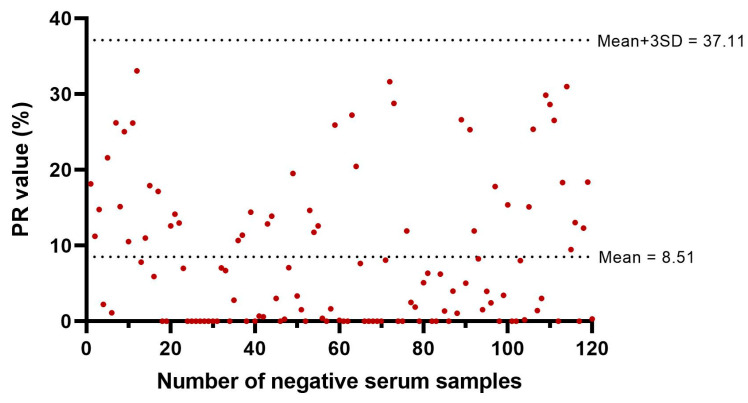
Dot plot of PR% values and mean and cutoff values of the TiLV-S4 ELISA for 120 fish not infected with TiLV.

To establish the cut-off value for the TiLV-S4 ELISA, 120 serum samples previously verified by TiLV-WV ELISA assays were utilized. The average PR value ($\overset{-}{x}$) of the negative samples was calculated as $\overset{-}{x}$ = 8.51, with a standard deviation (SD) of 9.53. The cut-off PR value was determined using the formula: PR value = $\overset{-}{x}$ + 3SD = 37.11. Samples with a PR value ≥ 37.11 were classified as TiLV-seropositive (Figure 3).

#### 3.2.4. Efficiency of TiLV-S4 ELISA Compared with the Reference Method

To evaluate the specificity and sensitivity of the TiLV S4-ELISA, a total of 80 serum samples were assessed. Prior to TiLV-S4 ELISA testing, all samples were analyzed by the TiLV WV-ELISA to confirm their TiLV serostatus. Forty serum samples were confirmed negative, and another 40 were positive, for TiLV antibodies by TiLV-WV ELISA. Using a cut-off PR value of 37.11%, the TiLV-S4 ELISA correctly identified 36 of the 40 TiLV-seronegative samples and 33 of the 40 TiLV-seropositive samples. Accordingly, the diagnostic specificity of the TiLV-S4 ELISA was 90.0% (36/40, 95% CI = 76.3 to 97.2%), and the diagnostic sensitivity was 82.5% (33/40, 95% CI =67.2 to 92.7%) compared to TiLV-WV ELISA results (Supplementary Table S1).

### 3.3. Pooling Efficiency

#### 3.3.1. Pooling Scenarios

**Figure 4:**
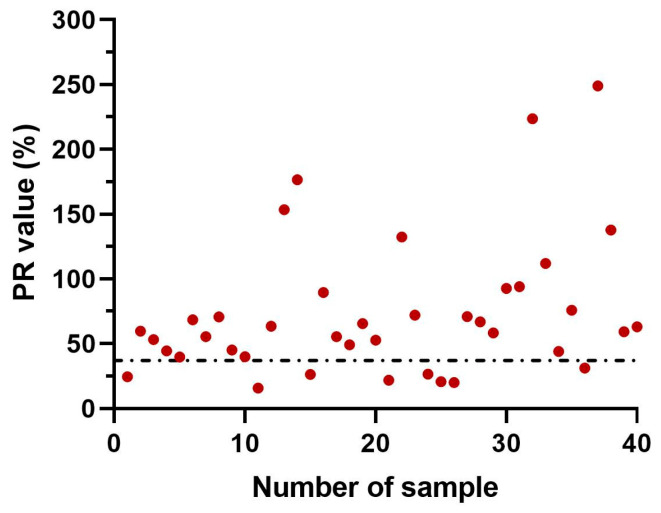
Detection of fish serum antibodies against TiLV in Farm A using TiLV-S4 ELISA. The dashed line indicates the assay’s cut-off value for seropositivity determination.

Of the 40 fish serum samples collected from farm A, which were taken 28 days after first mortality occurred, 32 samples (80%) were identified as TiLV-seropositive using the TiLV-S4 ELISA. The PR values for these positive samples ranged from 39.8% to 248.9%. In contrast, eight samples (20%) were below the cut-off PR value, with PR values ranging from 15.8% to 31.4%, and were classified as TiLV-seronegative (Figure 4).

To evaluate the effectiveness of using the TiLV-S4 ELISA with pooled serum samples for detecting antibodies against TiLV, various scenarios were tested using samples from farm A. When one TiLV-seropositive serum sample was added to a pool of five samples, all pooled samples tested negative (Table 3: sample no. 1–5). Similarly, adding two TiLV-seropositive serum samples resulted in only 1 of 11 pools (9.1%) testing positive (Table 3: sample no. 12), while the others remained negative. However, when three TiLV-seropositive serum samples were pooled, 11 out of 20 pools (55%) yielded positive results (Table 3: sample no. 17–36).

#### 3.3.2. Efficiency of Pooling Technique to Detect Antibodies Against TiLV in Fish Populations

To assess the efficiency of pooling for antibody surveillance in fish populations, serum samples with unknown serostatus from farms B, C, and D were pooled into groups of five samples each. A total of ten pooled samples were created from 40, 38, and 42 individual fish serum samples in farms B, C, and D, respectively. TiLV IgM antibodies were detected in 50% (5/10), 90% (9/10), and 20% (2/10) of pooled samples from farms B, C, and D, respectively (Table 4). The PR value of the pooled samples ranged from 41.0% to 192.0% (Table 4). Notably, each pooled sample contained between two and four individual TiLV-seropositive samples, with individual PR values ranging from 37.7% to 171.9%. In contrast, pools that were entirely seronegative or contained only one seropositive sample yielded false-negative results. The latter was observed in specific cases from Farm B (samples 3 and 5) and Farm D (sample 22).

The prevalence of TiLV-seropositive serum varied across farms, as shown in Figure S1. Farm A, with a cumulative mortality of 33% and samples collected 28 days post first mortality, had the highest seroprevalence of 80% (32/40 samples). Farm B, with 23% cumulative mortality and samples collected 39 days post first mortality, had a prevalence of 39.5% (15/38). Farm C, with 40% cumulative mortality and samples collected 60 days post first mortality, showed a seroprevalence of 45.2% (19/42). Farm D, with 40% cumulative mortality and samples collected 90 days post first mortality, had the lowest prevalence, at 15% (6/40) (Supplementary Figure S1).

### 3.4. Pooled Compared with Individual Test Results in Farms A, B, C, and D

In Farm A, which had one to three positive samples in each pool by design, the proportion of TP results was significantly greater when there were three positive samples (11/20) compared with two positive results (1/11) or one positive result (0/5). Fisher exact *p* values were 0.020 and 0.046, respectively. Among the 17 TP pools in farms B, C, and D, the range was one to four positive individual samples out of five tested, with a median of 2. Among the five FN pools in the same farms, the range was one to two positives, with a median of 1 (Table 5).

**Figure 5:**
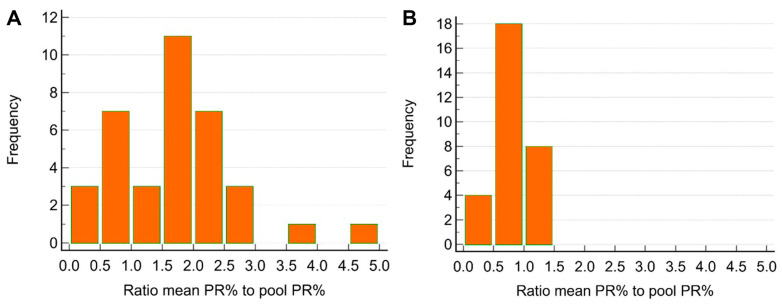
Ratio of the mean percentage reactivity (PR) of five individual serum samples to the pooled PR% of the component samples from (**A**) Farm A (*n* = 36) and (**B**) Farms B, C, and D (*n* = 30).

The ratio of the mean PR% to pooled PR% values varied in Farm A (*n* = 36) from 0.39 to 4.51, with a median of 1.76 (Figure 5A). In contrast to Farm A, ratio values in the other three farms (*n* = 30) were significantly lower (*p* < 0.001), with a median of 0.67 and a range from 0.33 to 1.27 (Figure 5B).

## 4. Discussion

Despite the relatively high prevalence of TiLV disease in tilapia world-wide [21,22], antigen-based or serological assays to support immunological studies and disease surveillance during TiLV outbreaks remain lacking. Measuring viral-specific antibodies is essential for estimating prevalence, tracking infection dynamics, and monitoring immune responses following infection or vaccination [14,15,16]. Our study introduces a sensitive and specific indirect ELISA capable of quantifying antibody response against TiLV in tilapia populations. For serological analysis in fish, ELISA is cost-effective, highly specific, and suitable for large-scale detection [11,12,13]. In contrast, molecular assays, which are widely used to detect TiLV in infected tissues, are effective [7,23,24], but they are limited in identifying fish that have survived infection. Notably, this limitation arises as the virus may have been cleared from the tissues or reduced to levels below the detection threshold of these assays [8]. Serological tests, especially ELISA, are a low-cost and practical alternative to direct detection tests for fish viruses, including TiLV, in countries where obtaining diagnostic samples is impractical or cost-prohibitive during disease outbreaks. Because blood collection is non-lethal, it is well suited to disease surveillance programs [25].

Previous studies have demonstrated the effectiveness of ELISA tests for assessing the immune response of red tilapia infected with TiLV under laboratory conditions, highlighting that antibody levels in virus-infected fish fluctuate following exposure [8]. Specifically, antibodies against TiLV can persist for over four months, suggesting that antibody-based assessments hold practical potential for disease diagnosis and provided a valuable diagnostic window [8]. However, the application of ELISA techniques to assess the immunological status of fish in aquaculture settings has not been extensively investigated. To address this gap, we employed an indirect ELISA using a recombinant protein derived from segment 4 of TiLV to detect specific antibodies against the virus. Indeed, the recombinant proteins have been widely utilized in ELISA assays for measuring antibody responses in fish serum or tissue following pathogen infection or vaccination [13,26,27,28]. The use of recombinant proteins offers distinct advantages, such as eliminating the need for pathogen culture, thereby simplifying the diagnostic process and reducing the associated cost. Furthermore, previous studies have successfully developed ELISA methods for detecting TiLV-specific antibodies in fish serum, mucus, and internal organs [9,10]. For example, Hu et al. [9] demonstrated the efficacy of an ELISA based on a recombinant protein from TiLV segment 8 for detecting specific antibodies in the serum of TiLV-infected fish from both laboratory and field samples. More recently, Lalruatfela et al. [10] developed an ELISA using a recombinant protein from TiLV segment 4, which demonstrated its effectiveness in detecting TiLV-specific antibodies in fish kidney and mucus samples. Although no significant antigenic variation among TiLV strains has been confirmed to date, recent studies have reported genetic divergence among TiLV isolates from different geographic regions, such as Vietnam and Israel, which may influence antibody recognition patterns [22]. Therefore, future studies should validate the TiLV-S4 ELISA using sera from fish infected with genetically diverse TiLV strains to ensure broad diagnostic applicability.

In this study, the cut-off value for the ELISA was determined using 120 TiLV-seronegative samples from TiLV-infected fish subjected to laboratory challenges. To ensure consistency, optical density (OD) values were calculated and presented as percentage reactivity (PR) values. The PR values were chosen over OD values to minimize the variability often observed when conducting ELISA across different plates. By calculating PR values as the percentage of positive ELISA results relative to extinction of negative serum on each plate or across multiple plates, variability is reduced [11]. The use of PR values has become increasingly popular in recent ELISA development studies [29,30]. Furthermore, the repeatability and reproducibility of the TiLV-S4 ELISA were assessed, showing that intra-assay and inter-assay variations were below 10%, which demonstrates the repeatability and reproducibility of the assay [31].

The TiLV-S4 ELISA developed in this study was validated against the previously established TiLV WV-ELISA [8], demonstrating a diagnostic sensitivity of 82.5% and specificity of 90%, with minimal false positives. The WV-ELISA uses a whole-virus antigen, which presents multiple viral epitopes from both structural and non-structural proteins, allowing it to detect a broader range of antibody responses. In contrast, the TiLV-S4 ELISA detects antibodies specific to a single structural protein, which is the nucleoprotein encoded by segment 4. Therefore, if the immune response in some fish is primarily directed against other viral components, the TiLV-S4 ELISA may yield false-negative results. However, the specificity and consistency of antibody responses to immunodominant antigens of TiLV remain to be fully characterized. Similar recombinant protein-based ELISAs have shown comparable performance, such as the TiLV-S8 ELISA, with 100% sensitivity and 92.6% specificity [9], and a TiLV segment 4 ELISA, with 82.4% sensitivity and 100% specificity, against RT-PCR [10]. These results highlight the reliability of TiLV-S4 ELISA and its potential for effective disease surveillance in tilapia populations. Indeed, the TiLV-S4 ELISA was shown to be an effective tool for assessing serostatus of fish populations following a TiLV outbreak. Notably, analysis of the prevalence of TiLV-specific antibodies in surviving fish at various time points revealed that antibody titers can persist for over three months, although prevalence declines over time. The sustained immune response is consistent with previous studies that reported the presence of TiLV-specific antibodies up to four months post-infection [8]. Importantly, the prevalence of TiLV-seropositive fish was associated with higher cumulative mortality and the duration of seroreactivity following the outbreak.

Testing individual fish serum samples can be costly and time-consuming, making the pooling technique a more cost-effective and efficient alternative for laboratory workflows [17,18,19]. Despite its advantage, research on the effects of pooled samples on ELISA performance in aquatic animals remains limited compared to the extensive studies on terrestrial animals [32,33]. In this study, the TiLV-S4 ELISA showed no false-positive results when testing pooled seronegative samples, and the median ratio of mean PR% to pooled PR% from Farms B, C, and D was consistent and close to 1, which indicates strong agreement between pooled and individual results. We selected a pool size of five samples, as it provides a practical balance between cost-efficiency and diagnostic sensitivity, as supported by our preliminary tests. Pooling serum samples combined with the TiLV-S4 ELISA increased the detection of seropositive fish by covering a broader population at reduced cost, particularly when pools contained at least two or three seropositive samples. However, some pooled samples yielded false-negative results due to the dilution of positive sera within the pool. Similar limitations were observed in previous studies [10], where pooled kidney and mucus samples produced false negatives, which highlight the sensitivity constraints of pooling. In Farm A, a higher false-negative rate was found, likely due to the collection of samples from a population recently affected by a disease outbreak. These samples had high antibody titers and a high prevalence of seropositive sera, which may have caused antibody aggregation and clumping, which disrupt ELISA performance by impairing antibody binding and reducing assay sensitivity [34,35]. These findings emphasize the need to consider the composition of pooled samples and the potential trade-offs in analytical sensitivity when using this approach. Additionally, pooling of serum samples, as studied in the present research, is most likely to be useful for low-prevalence scenarios (e.g., one, two, or perhaps three positive samples in a pool of 5). If prevalence is high (e.g., four or five positive samples per pool), pooling would provide minimal efficiency gain over individual testing for determining population-level infection status (i.e., presence or absence of TiLV). Therefore, we did not include combinations with four or five positive sera in our pooling experiments. Future studies should also include defined serial dilutions of seropositive serum within pooled samples to better understand the detection threshold and quantitative relationship between antibody concentration and ELISA signal in pooled testing.

The application of the pooling technique with the TiLV-S4 ELISA demonstrated its potential as a cost-effective and efficient strategy for detecting TiLV antibodies at the farm level. Specifically, data from Farms B, C, and D validated the approach by testing 10 pools of serum, each comprising samples from five fish, alongside their corresponding individual samples. This method significantly reduced the number of tests required from 50 individual tests to just 10 pooled tests, with minimal additional handling and laboratory resource usage. However, the small sample size across the three farms suggests caution when extrapolating these findings to larger populations. Despite this limitation, our results highlight the practicality of pooling for large-scale surveillance of TiLV, particularly in resource-limited settings, and provides a basis for further refinement and implementation of this method in aquaculture diagnostics. Moreover, an ELISA may be applied as part of an integrated diagnostic approach, in conjunction with outbreak timing, clinical signs, and mortality patterns. Based on our observations, fish from recent and severe outbreaks (e.g., Farm A) often showed high antibody levels that made pooled ELISA results comparable to individual testing. In contrast, in farms with outbreaks occurring more than 1–3 months prior (e.g., Farms B, C, and D), declining antibody levels in surviving fish made pooling a more practical and cost-effective strategy for population-level surveillance.

## 5. Conclusions

Taken together, our study introduces and optimizes an indirect ELISA based on recombinant TiLV segment 4 protein, which offers a reliable and effective tool for detecting TiLV antibodies in tilapia. It also highlights the practicality of pooling serum samples as a cost-effective and efficient diagnostic strategy for large-scale surveillance. However, the sensitivity of the pooling technique depends on infection prevalence, indicating the need for careful planning and consideration during implementation. These findings present a valuable diagnostic tool and strategy for improving TiLV detection methods and highlight the potential of ELISA-based approaches in managing TiLV control and advancing tilapia health management.

## Figures and Tables

**Table 1:** Details for sampling blood collection from farms with a history of a TiLV outbreak.

Farms	Total Sample	Cumulative Mortality (%) During Outbreak	Day Post Infection (dpi)	Outbreak Start Date	Blood Collection Date	Average Fish Weight (g)
A	40	33	28	10 September 2022	7 October 2022	150
B	38	23	39	28 August 2022	7 October 2022	30
C	42	40	60	6 July 2022	9 September 2022	200
D	40	40	90	6 July 2022	7 October 2022	400

**Table 2:** Estimates of coefficients of variation (CV) from six samples.

	Samples	Mean OD Value	SD	CV%
Repeatability (Intra-assay)	1	1.06	0.04	3.6
	2	0.91	0.01	1.1
	3	1.47	0.13	8.7
	4	0.08	0.00	2.7
	5	0.17	0.00	2.7
	6	0.21	0.02	7.7
Reproducibility (Inter-assay)	1	1.03	0.08	7.7
	2	0.91	0.03	3.1
	3	1.45	0.11	7.6
	4	0.08	0.01	6.8
	5	0.18	0.01	5.0
	6	0.20	0.00	1.7

Positive serum samples are indicated by shaded boxes.

**Table 3:** The pooled PR values of five serum samples, including individual component samples from farm A with one, two, or three TiLV-seropositive individuals.

Sample No.	PR Value (%) of Pooled Sample	PR Value (%) of Individual Sample				
1	14.2	223.6	26.4	21.9	26.5	20.8
2	20.8	94.0	21.9	26.5	20.8	20.1
3	14.2	55.4	15.8	26.4	21.9	26.5
4	16.5	49.2	26.4	21.9	26.5	20.8
5	12.3	44.0	21.9	26.5	20.8	20.1
6	14.9	40.1	39.7	15.8	26.4	24.6
7	29.4	176.5	44.0	20.1	31.4	24.6
8	16.2	70.9	40.1	15.8	26.4	21.9
9	26.8	67.0	44.0	20.8	20.1	26.5
10	21.1	53.4	44.0	20.1	20.8	31.4
11	26.0	45.2	44.0	20.1	31.4	24.6
12	131.2	248.9	223.6	21.9	26.5	20.8
13	35.1	153.5	67.0	15.8	26.4	21.9
14	16.2	176.5	58.5	26.5	20.8	20.1
15	20.4	70.9	63.2	26.4	21.9	26.5
16	17.1	45.2	44.5	21.9	26.5	20.8
17	16.9	44.5	39.7	40.1	15.8	26.4
18	25.7	59.7	39.7	40.1	31.4	24.6
19	13.4	52.8	39.7	40.1	24.6	15.8
20	37.7	58.5	55.4	45.2	21.9	26.5
21	15.5	59.4	49.2	39.7	31.4	24.6
22	95.8	59.7	53.4	44.5	26.4	20.8
23	22.9	63.5	45.2	40.1	15.8	26.4
24	23.9	72.0	55.4	44.0	20.8	20.1
25	54.5	76.0	58.5	45.2	24.6	15.8
26	17.5	76.0	44.0	39.7	24.6	31.4
27	72.5	89.6	68.5	55.4	20.8	20.1
28	137.5	94.0	70.9	63.5	15.8	26.4
29	60.1	92.7	70.9	63.2	21.9	26.5
30	24.6	112.0	39.7	40.1	15.8	26.4
31	133.3	153.5	58.5	49.2	31.4	24.6
32	27.4	153.5	44.5	44.0	31.4	24.6
33	80.4	176.5	89.6	72.0	26.4	21.9
34	39.0	223.6	176.5	44.5	26.5	20.8
35	87.3	248.9	39.7	40.1	24.6	15.8
36	157.5	248.9	94.0	44.0	20.1	31.4

PR values above the negative cut-off (37.11) are indicated by shaded boxes.

**Table 4:** PR value (%) of pooled samples of five tilapia and the corresponding individual serum values from farms B, C, and D.

Sample No.	Farm	PR Value (%) of Pooled Sample	PR Value (%) of Individual Sample				
1	B	41.5	60.2	63.5	43.9	11.5	13.6
2		49.2	71.6	79.7	12.5	18.1	25.2
3		26.4	60.1	29.1	15.1	29.0	8.0
4		47.5	104.2	77.0	30.5	25.5	18.7
5		19.2	40.0	24.7	13.0	31.4	7.9
6		26.2	48.6	70.4	31.9	3.6	12.6
7		84.0	57.6	75.4	47.4	41.2	31.5
8		30.2	71.6	43.9	15.1	14.3	12.9
9		40.9	104.2	48.6	57.6	29.0	7.9
10		38.1	79.7	11.5	12.9	29.1	30.5
11	C	83.5	101.1	48.4	19.2	27.7	34.7
12		146.5	63.7	160.8	128.4	27.0	27.2
13		114.5	162.9	46.7	37.7	32.7	13.5
14		16.3	18.1	2.9	12.1	16.1	24.1
15		103.9	171.9	41.9	29.4	17.4	32.6
16		192.0	60.3	68.5	142.8	27.1	13.8
17		94.9	113.1	82.2	97.5	99.7	23.7
18		66.5	101.6	15.5	24.7	35.9	23.8
19		166.7	91.3	63.7	162.9	101.1	11.5
20		89.0	113.1	60.3	18.1	32.6	23.8
21	D	14.0	8.5	8.6	19.5	24.5	9.7
22		22.3	64.8	19.5	17.4	22.5	3.7
23		27.9	10.1	16.6	18.6	29.0	35.7
24		32.7	14.1	8.0	18.4	29.9	27.5
25		12.6	6.4	2.3	1.7	7.7	7.3
26		21.1	11.5	7.4	8.4	13.9	13.6
27		100.7	95.0	123.6	32.5	16.3	30.8
28		94.5	82.6	53.3	43.2	15.0	34.4
29		14.8	8.5	3.7	10.1	2.3	6.4
30		23.4	11.5	30.8	15.0	8.6	19.5

PR values above the negative cut-off (37.11) are indicated by shaded boxes.

**Table 5:** The frequency of TP, TN, and FN pools varied significantly among farms (chi-square *p* value < 0.0001).

Farms	Frequency			Total Pools
	True-Positive Pools (TP)	True-Negative Pools (TN)	False-Negative Pools (FN)	
A	12	Not applicable	24	36
B	6	0	4	10
C	9	1	0	10
D	2	7	1	10

## Data Availability

The data presented in this study are available on request from the corresponding author. The data are not publicly available due to the anonymity granted to all participating parties.

## Supplementary Materials

The following supporting information can be downloaded at: https://www.mdpi.com/article/10.3390/v17070877/s1, Figure S1: PR value (%) of individual serum samples in Farms A, B, C, and D using TiLV-S4 ELISA. Table S1. Optical density (OD) and percent reactivity (PR) values obtained from the TiLV-WV-ELISA and TiLV-S4-ELISA for 40 seronegative and 40 seropositive tilapia serum samples, used to evaluate the diagnostic sensitivity and specificity of the TiLV ELISA assays.

## References

[1] FAO (2022). The State of World Fisheries and Aquaculture (SOFIA). Towards Blue Transformation [Internet].

[2] WOAH (2022). Infection with Tilapia Lake Virus (TiLV)—A Novel Orthomyxo-like Virus. WOAH Disease Card [Internet].

[3] Surachetpong W., Roy S.R.K., Nicholson P. (2020). Tilapia lake virus: The story so far. J. Fish Dis..

[4] Jaemwimol P., Rawiwan P., Tattiyapong P., Kamlangdee A., Surachetpong W. (2018). Susceptibility of important warm water fish species to tilapia lake virus (TiLV) infection. Aquaculture.

[5] Phusantisampan T., Tattiyapong P., Mutrakulcharoen P., Sriariyanun M., Surachetpong W. (2019). Rapid detection of tilapia lake virus using a one-step reverse transcription loop-mediated isothermal amplification assay. Aquaculture.

[6] Piewbang C., Tattiyapong P., Techangamsuwan S., Surachetpong W. (2021). Tilapia lake virus immunoglobulin G (TiLV IgG) antibody: Immunohistochemistry application reveals cellular tropism of TiLV infection. Fish Shellfish Immunol..

[7] Tattiyapong P., Sirikanchana K., Surachetpong W. (2018). Development and validation of a reverse transcription quantitative polymerase chain reaction for tilapia lake virus detection in clinical samples and experimentally challenged fish. J. Fish Dis..

[8] Tattiyapong P., Dechavichitlead W., Waltzek T.B., Surachetpong W. (2020). Tilapia develop protective immunity including a humoral response following exposure to tilapia lake virus. Fish Shellfish Immunol..

[9] Hu H., Zeng W., Wang Y., Bergmann S.M., Yin J. (2020). Development and application of a recombinant protein-based indirect ELISA for detection of anti-tilapia lake virus IgM in sera from tilapia. Aquaculture.

[10] Lalruatfela, Bedekar M.K., Godavarikar A., Valsalam A., Gireesh Babu P., Rajendran K.V. (2024). Molecular cloning and expression of codon-optimized segment 4 hypothetical protein (35 kDa) of tilapia lake virus (TiLV) in pET-28a( +) expression vector and development of indirect ELISA test. Aquac. Int..

[11] Bergmann S.M., Wang Q., Zeng W., Li Y., Wang Y., Matras M. (2017). Validation of a KHV antibody enzyme-linked immunosorbent assay (ELISA). J. Fish Dis..

[12] Kim H.J., Park J.S., Kwon S.R. (2015). Development of a stringent ELISA protocol to evaluate anti-viral hemorrhagic septicemia virus-specific antibodies in olive flounder *Paralichthys olivaceus* with improved specificity. J. Microbiol..

[13] Zeng W., Wang Y., Guo Y., Bergmann S.M., Yin Y., Li Y. (2018). Development of a VP38 recombinant protein-based indirect ELISA for detection of antibodies against grass carp reovirus genotype II (iELISA for detection of antibodies against GCRV II). J. Fish Dis..

[14] Kim H.J., Oseka N., Nishizawa T., Yoshimizu M. (2009). Protection of rainbow trout from infectious hematopoietic necrosis (IHN) by injection of infectious pancreatic necrosis virus (IPNV) or poly(I:C). Dis. Aquat. Organ..

[15] Nishizawa T., Takami I., Kokawa Y., Yoshimizu M. (2009). Fish immunization using a synthetic double-stranded RNA Poly(I:C), an interferon inducer, offers protection against RGNNV, a fish nodavirus. Dis. Aquat. Organ..

[16] Takami I., Kwon S.R., Nishizawa T., Yoshimizu M. (2010). Protection of Japanese flounder *Paralichthys olivaceus* from viral hemorrhagic septicemia (VHS) by Poly(I:C) immunization. Dis. Aquat. Organ..

[17] Fereidouni S.R., Harder T.C., Gaidet N., Ziller M., Hoffmann B., Hammoumi S. (2012). Saving resources: Avian influenza surveillance using pooled swab samples and reduced reaction volumes in real-time RT-PCR. J. Virol. Methods.

[18] Johnson S.J., Hick P.M., Robinson A.P., Rimmer A.E., Tweedie A., Becker J.A. (2019). The impact of pooling samples on surveillance sensitivity for the megalocytivirus Infectious spleen and kidney necrosis virus. Transbound. Emerg. Dis..

[19] Nguyen N.T., Bish E.K., Bish D.R. (2021). Optimal pooled testing design for prevalence estimation under resource constraints. Omega.

[20] Yamkasem J., Roy S.R.K., Khemthong M., Gardner I.A., Syrachetpong W. (2021). Diagnostic sensitivity of pooled samples for the detection of tilapia lake virus and application to the estimation of within-farm prevalence. Transbound. Emerg. Dis..

[21] Kembou-Ringert J.E., Steinhagen D., Readman J., Daly J.M., Adamek M. (2023). Tilapia Lake Virus Vaccine Development: A Review on the Recent Advances. Vaccines.

[22] Tran T.H., Nguyen V.T.H., Bui H.C.N., Tran Y.B.T., Tran H.T.T., Le T.T.T. (2022). Tilapia Lake Virus (TiLV) from Vietnam is genetically distantly related to TiLV strains from other countries. J. Fish Dis..

[23] Dong H., Siriroob S., Meemetta W., Santimanawong W., Gangnonngiw W., Pirarat N. (2017). Emergence of tilapia lake virus in Thailand and an alternative semi-nested RT-PCR for detection. Aquaculture.

[24] Eyngor M., Zamostiano R., Kembou Tsofack J.E., Berkowitz A., Bercovier H., Tinman S. (2014). Identification of a novel RNA virus lethal to tilapia. J. Clin. Microbiol..

[25] Jaramillo D., Peeler E.J., Laurin E., Gardner I.A., Whittington R.J. (2017). Serology in Finfish for Diagnosis, Surveillance, and Research: A Systematic Review. J. Aquat. Anim. Health.

[26] Matsuyama T., Sano N., Takano T., Sakai T., Yasuike M., Fujiwara A. (2018). Antibody profiling using a recombinant protein-based multiplex ELISA array accelerates recombinant vaccine development: Case study on red sea bream iridovirus as a reverse vaccinology model. Vaccine.

[27] Rahnama R., Peyghan R., Reza Seyfi Abad Shapouri M., Rezaie A., Shahbazian N. (2019). Designing an in-house ELISA to detect antibody against viral haemorrhagic septicaemia virus using recombinant N protein in Iranian farmed rainbow trout (*Oncorhynchus mykiss*). Aquac. Res..

[28] Watanabe K.I., Nishizawa T., Yoshimizu M. (2000). Selection of brood stock candidates of barfin flounder using an ELISA system with recombinant protein of barfin flounder nervous necrosis virus. Dis. Aquat. Organ..

[29] Dong B., Zhang G., Zhang X., Chen X., Zhang M., Li L. (2021). Development of an Indirect ELISA Based on Spike Protein to Detect Antibodies against Feline Coronavirus. Viruses.

[30] Lu M., Liu Q., Wang X., Zhang J., Zhang X., Shi D. (2020). Development of an indirect ELISA for detecting porcine deltacoronavirus IgA antibodies. Arch. Virol..

[31] Jacobson R.H. (1998). Validation of serological assays for diagnosis of infectious diseases. Rev. Sci. Tech..

[32] Baruch J., Suanes A., Piaggio J.M., Gil A.D. (2020). Analytic Sensitivity of an ELISA Test on Pooled Sera Samples for Detection of Bovine Brucellosis in Eradication Stages in Uruguay. Front. Vet. Sci..

[33] Hernandez-Medrano J.H., Espinosa-Castillo L.F., Rodriguez A.D., Gutierrez C.G., Wapenaar W. (2021). Use of pooled serum samples to assess herd disease status using commercially available ELISAs. Trop. Anim. Health Prod..

[34] Nezlin R. (1998). The Immunoglobulins: Structure and Function.

[35] Wang W., Nema S., Teagarden D. (2010). Protein aggregation-pathways and influencing factors. Int. J. Pharm..

