# Supplementary material for: Assessment and Performance of Pooled Serum Samples for Monitoring Farm-Level Immunity in Tilapia Infected with Tilapia Lake Virus
Source: Viruses. 2025 Jun 22;17(7):877. doi: 10.3390/v17070877 (PMC12300263; doi:10.3390/v17070877)
Supplement: Supplementary file 1 [file viruses-17-00877-s001.zip › viruses-3673082-supplementary.pdf]

Supplementary Table S1. Optical density (OD) and percent reactivity (PR) values obtained from the TiLV-WV-ELISA and TiLV-S4-ELISA for 40 seronegative and 40 seropositive tilapia serum samples, used to evaluate the diagnostic sensitivity and specificity of the TiLV ELISA assays.

**TiLV-WV-ELISA**				**TiLV-S4-ELISA**			
Seronegative sample		Seropositive sample		Seronegative sample		Seropositive sample	
ID	OD value	ID	OD value	ID	PR value	ID	PR value
Seronegative 1	0.14	Seropositive 1	1.93	Seronegative 1	0.00	Seropositive 1	82.79
Seronegative 2	0.49	Seropositive 2	1.07	Seronegative 2	0.00	Seropositive 2	46.99
Seronegative 3	0.52	Seropositive 3	1.05	Seronegative 3	2.82	Seropositive 3	38.81
Seronegative 4	0.13	Seropositive 4	1.29	Seronegative 4	0.00	Seropositive 4	77.23
Seronegative 5	0.44	Seropositive 5	2.11	Seronegative 5	0.00	Seropositive 5	120.34
Seronegative 6	0.22	Seropositive 6	1.45	Seronegative 6	0.00	Seropositive 6	286.70
Seronegative 7	0.40	Seropositive 7	1.46	Seronegative 7	1.63	Seropositive 7	149.73
Seronegative 8	0.59	Seropositive 8	2.09	Seronegative 8	1.99	Seropositive 8	166.51
Seronegative 9	0.36	Seropositive 9	2.43	Seronegative 9	0.00	Seropositive 9	97.26
Seronegative 10	0.17	Seropositive 10	2.33	Seronegative 10	0.00	Seropositive 10	159.19
Seronegative 11	0.56	Seropositive 11	1.74	Seronegative 11	0.00	Seropositive 11	310.92
Seronegative 12	0.07	Seropositive 12	2.53	Seronegative 12	0.00	Seropositive 12	42.88
Seronegative 13	0.25	Seropositive 13	1.68	Seronegative 13	7.82	Seropositive 13	22.50
Seronegative 14	0.59	Seropositive 14	1.64	Seronegative 14	97.14	Seropositive 14	52.62
Seronegative 15	0.36	Seropositive 15	1.60	Seronegative 15	10.99	Seropositive 15	19.13
Seronegative 16	0.47	Seropositive 16	1.44	Seronegative 16	17.92	Seropositive 16	41.12
Seronegative 17	0.50	Seropositive 17	1.68	Seronegative 17	66.51	Seropositive 17	332.32
Seronegative 18	0.33	Seropositive 18	1.29	Seronegative 18	49.88	Seropositive 18	12.68
Seronegative 19	0.38	Seropositive 19	1.14	Seronegative 19	17.14	Seropositive 19	8.33
Seronegative 20	0.52	Seropositive 20	1.42	Seronegative 20	6.69	Seropositive 20	17.88
Seronegative 21	0.33	Seropositive 21	1.20	Seronegative 21	0.00	Seropositive 21	24.02
Seronegative 22	0.22	Seropositive 22	1.01	Seronegative 22	0.00	Seropositive 22	13.50
Seronegative 23	0.51	Seropositive 23	1.51	Seronegative 23	14.40	Seropositive 23	119.84

Seronegative 24	0.52	Seropositive 24	1.16	Seronegative 24	0.00	Seropositive 24	101.92
Seronegative 25	0.46	Seropositive 25	1.70	Seronegative 25	0.70	Seropositive 25	125.74
Seronegative 26	0.53	Seropositive 26	1.79	Seronegative 26	1.53	Seropositive 26	164.20
Seronegative 27	0.29	Seropositive 27	1.11	Seronegative 27	0.00	Seropositive 27	75.08
Seronegative 28	0.39	Seropositive 28	1.31	Seronegative 28	54.77	Seropositive 28	121.87
Seronegative 29	0.36	Seropositive 29	1.00	Seronegative 29	11.74	Seropositive 29	175.08
Seronegative 30	0.61	Seropositive 30	1.82	Seronegative 30	0.39	Seropositive 30	92.02
Seronegative 31	0.38	Seropositive 31	0.94	Seronegative 31	0.00	Seropositive 31	69.84
Seronegative 32	0.66	Seropositive 32	1.55	Seronegative 32	1.64	Seropositive 32	176.60
Seronegative 33	0.51	Seropositive 33	1.71	Seronegative 33	25.90	Seropositive 33	139.44
Seronegative 34	0.37	Seropositive 34	1.79	Seronegative 34	0.00	Seropositive 34	80.83
Seronegative 35	0.31	Seropositive 35	1.35	Seronegative 35	0.00	Seropositive 35	114.63
Seronegative 36	0.69	Seropositive 36	1.31	Seronegative 36	27.23	Seropositive 36	119.37
Seronegative 37	0.64	Seropositive 37	1.33	Seronegative 37	7.63	Seropositive 37	184.27
Seronegative 38	0.57	Seropositive 38	1.25	Seronegative 38	0.00	Seropositive 38	178.56
Seronegative 39	0.50	Seropositive 39	1.22	Seronegative 39	0.00	Seropositive 39	87.60
Seronegative 40	0.58	Seropositive 40	1.50	Seronegative 40	8.06	Seropositive 40	126.76

False positive results (defined as seronegative samples with PR values exceeding the cut-off of 37.11) are indicated in red text, while false negative results (seropositive samples with PR values below the cut-off) are shaded in grey.

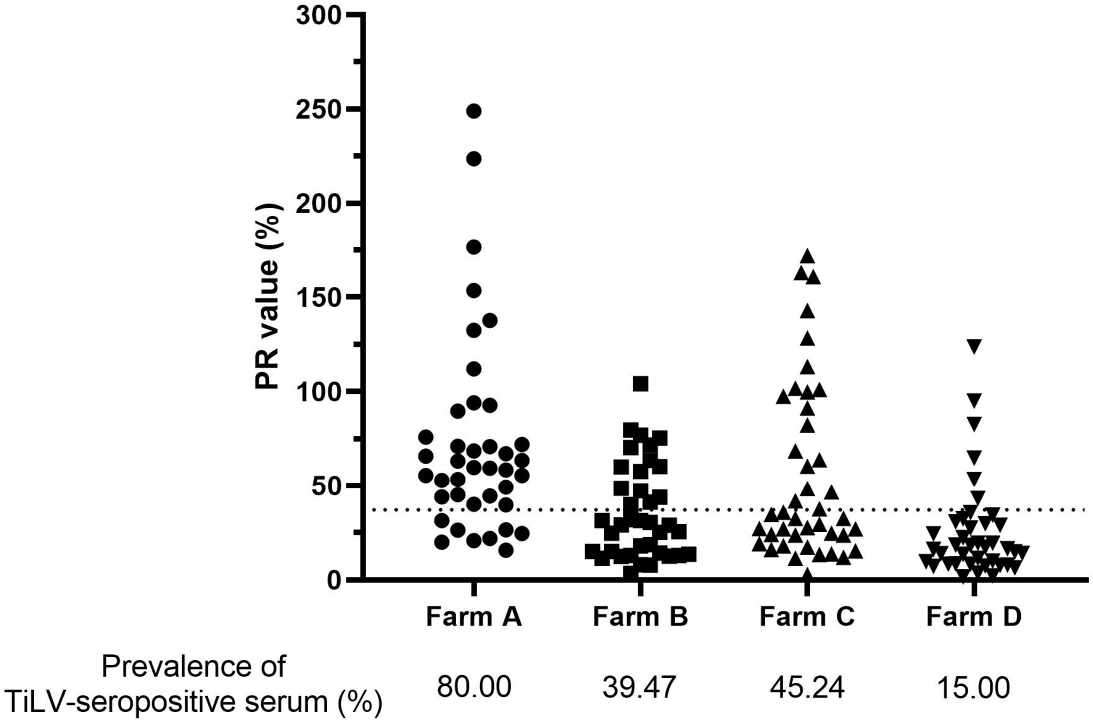

Supplementary Figure S1. Percent reactivity (PR%) values of individual serum samples from Farm A, B, C, and D, as measured by the TiLV-S4 ELISA.
